# Rethinking Employer Communication and Support for Home Care Aides: A Case Study of Pandemic Group Support Calls

**DOI:** 10.1093/geroni/igab046.838

**Published:** 2021-12-17

**Authors:** Emma Tsui

**Affiliations:** CUNY Graduate School of Public Health & Health Policy, New York, New York, United States

## Abstract

This case study explores an employer-initiated biweekly group support call for home care aides implemented by a large New York City-based home care agency during the COVID-19 pandemic. Specifically, we investigate how agency staff used information gathered through these calls to intervene into existing agency communication and support systems for aides. Our single-site case study analyzes detailed notes from almost 100 support calls that took place between April 2020 and March 2021, as well as interviews with agency staff from communications, human resources, nursing, and other departments that support aides. We compare and contrast new communication and support mechanisms advanced in conjunction with these calls with agency systems pre-pandemic. Our findings suggest that while calls were initially targeted toward providing emotional and operational support, staff also advocated for more systemic supports. We discuss the sustainability of these new efforts, as well as ongoing barriers and gaps.

